# 

**Published:** 1884-04

**Authors:** 


### CONTENTS.

### COMMUNICATIONS.

Phosphate of Lime and the Teeth........ 163
Carbolic Acid........ 173

### PROCEEDINGS.

Fortieth Annual Meeting Miss. Valley Dental Association........ 184

### SELECTIONS.

Salmagundi........ 208

### PROPOSITIONS.

A Proposition........ 212

### EDITORIAL.

The S uthern Dental Association........ 213
Mad River Dental Society........ 214
Dental Society State of New York........ 214

